# From Awareness to Social Action: The Role of Dementia Friends in Sustaining Dementia Inclusiveness

**DOI:** 10.1093/geroni/igab046.1981

**Published:** 2021-12-17

**Authors:** Bonnie Burman, Martha Williman

**Affiliations:** Ohio Council for Cognitive Health, New Albany, Ohio, United States

## Abstract

According to the World Dementia Council, three components are important to effectively engage a community to become dementia inclusive, 1) raising awareness and consequently decreasing stigma, 2) enabling participation, and 3) providing support—including in health and care settings. Too many times these components are separate initiatives thus limiting their effectiveness and sustainability. By applying the collective impact model and utilizing the Dementia Friends program as the link between the three, all dementia inclusive efforts can be enhanced and sustained regardless of the range of activities and approaches a community chooses to adopt. This symposium provides both evidence and examples of how to personalize and employ the Dementia Friends program to optimize the process, outcome, and impact of dementia inclusive initiatives. By engaging the entire community, awareness is raised, the structure is in place to enable action, and cross-sector collaboration will ensure continuation and sustainability of these important efforts.

